# Concurrent Clade I and Clade II Monkeypox Virus Circulation, Cameroon, 1979–2022 

**DOI:** 10.3201/eid3003.230861

**Published:** 2024-03

**Authors:** Delia D. Djuicy, Serge A. Sadeuh-Mba, Chanceline N. Bilounga, Martial G. Yonga, Jules B. Tchatchueng-Mbougua, Gael D. Essima, Linda Esso, Inès M.E. Nguidjol, Steve F. Metomb, Cornelius Chebo, Samuel M. Agwe, Placide A. Ankone, Firmin N.N. Ngonla, Hans M. Mossi, Alain G.M. Etoundi, Sara I. Eyangoh, Mirdad Kazanji, Richard Njouom

**Affiliations:** Centre Pasteur of Cameroon, Yaounde, Cameroon (D.D. Djuicy, S.A. Sadeuh-Mba, M.G. Yonga, J.B. Tchatchueng-Mbougua, G.D. Essima, S.I. Eyangoh, M. Kazanji, R. Njouom);; Ministry of Public Health, Yaounde (C.N. Bilounga, L. Esso, I.M.E. Nguidjol, S.F. Metomb, C. Chebo, S.M. Agwe, P.A. Ankone, F.N.N. Ngonla, H.M. Mossi, A.G.M. Etoundi);; University of Douala, Cameroon (C.N. Bilounga);; University of Bamenda, Cameroon (L. Esso)

**Keywords:** Mpox, monkeypox virus, MPXV, viruses, zoonoses, surveillance, surveillance system, epidemiology, clade I, clade II, concurrent circulation, geographic segregation, forest, Cameroon, Central Africa, West Africa

## Abstract

During 1979–2022, Cameroon recorded 32 laboratory-confirmed mpox cases among 137 suspected mpox cases identified by the national surveillance network. The highest positivity rate occurred in 2022, indicating potential mpox re-emergence in Cameroon. Both clade I (n = 12) and clade II (n = 18) monkeypox virus (MPXV) were reported, a unique feature of mpox in Cameroon. The overall case-fatality ratio of 2.2% was associated with clade II. We found mpox occurred only in the forested southern part of the country, and MPXV phylogeographic structure revealed a clear geographic separation among concurrent circulating clades. Clade I originated from eastern regions close to neighboring mpox-endemic countries in Central Africa; clade II was prevalent in western regions close to West Africa. Our findings suggest that MPXV re-emerged after a 30-year lapse and might arise from different viral reservoirs unique to ecosystems in eastern and western rainforests of Cameroon.

Monkeypox virus (MPXV) is an emerging zoonotic *Orthopoxvirus* causing mpox in humans, a disease similar to the eradicated smallpox (*1*). Since identification in a monkey in 1958 (*2*) and a human in 1970 (*3*), MPXV-associated outbreaks have occurred primarily in rural rainforests in countries of Central and West Africa (*4*–*6*).

Mpox is characterized by an influenza-like syndrome accompanied by adenopathy and maculopapular rashes typically developing on the palms of the hands and soles of the feet (*4*,*7*). For infected persons, supportive care and antiviral treatments, including cidofovir and tecovirimat, are provided (*4*). Cross-immunity with smallpox vaccination and a new generation of smallpox vaccines equally offer some protection (*8*–*10*). However, after smallpox vaccination was discontinued in the early 1980s, herd immunity gradually declined, enabling re-emergence of mpox, which is highlighted by the increased number of cases in Africa during the past 3 decades (*4*,*8*,*11*–*13*). Since early 2022, case counts have surged, and ≈1,215 confirmed mpox cases and 219 deaths were reported in Africa by December 28, 2022 (*14*). Before April 2022, mpox cases in the Western Hemisphere were typically reported from exposure to the exotic pet trade and international travel (*15*–*20*). Since then, MPXV-associated outbreaks have occurred worldwide, affecting >100 countries outside Africa (*4*,*21*) and becoming a global public health concern.

Primary MPXV transmission can occur through direct contact with body fluids or skin lesions of infected animals or indirectly via contaminated fomites. Similar contact with an infected person or with infected respiratory droplets might also lead to human-to-human secondary transmission, the main transmission mode of the 2022 global outbreak (*4*,*22*). Historically, primary zoonotic transmission was more common and mostly involved an at-risk population of hunters, butchers, and bushmeat handlers; secondary transmission was rare, but nosocomial and household transmission have been described (*3*,*13*,*23*–*25*).

Phylogenetic studies report 2 distinct MPXV clades: clade I, prevalent in Central Africa, and clade II, endemic to West Africa (*5*,*6*,*26*–*28*). However, Cameroon is an exception, and both clades concurrently circulate in the country (*6*,*29*). Clade I is further subdivided into lineages 1–5 and clade II into subclades IIa and IIb; clade IIb is responsible for the multicountry outbreak that began in 2022 (*27*,*28*,*30*). Globally, MPXV lethality rates vary from 1% to 10%, and clade I is known to have higher mortality rates than clade II (*4*,*24*,*25*). The MPXV animal reservoir has not yet been identified, but the virus can infect a wide range of mammals, and *Funisciurus* squirrels and *Graphiurus lorraineus* mice are thought to be the most probable MPXV reservoirs (*31*–*33*).

In Cameroon, only 4 confirmed mpox cases were documented before the 2022 outbreak, 1 in each 1979, 1980, 1989, and 30 years later in 2018 (*29*,*34*–*36*). According to public health reports, more cases could have occurred and been undocumented in the country, particularly during 2018–2021, and especially in 2022, during which an mpox outbreak of unprecedented magnitude occurred and had recurrent clusters of cases (*37*). However, whether those infections were associated with importations from neighboring countries or from occurrence of indigenous primary or secondary transmission remains unclear (*29*). Overall, data on the epidemiologic features of MPXV occurrence and transmission dynamics in Cameroon are scarce. We investigated the clinical, epidemiologic, and molecular features of MPXV-associated outbreaks in Cameroon.

## Methods

### Sample Location

Cameroon is in central Africa and is divided into 10 administrative regions. Cameroon is known as Africa in miniature for its diverse agroecologic background: the steppe and savanna in the Far North, North, and Adamawa regions; the coastal zones in the Littoral and Southwest regions; mountain highlands in the Northwest and West regions; and the rainforest in the Centre, South, Southwest, and East regions (*38*). Cameroon has 3 major tropical forests: the Congo Basin Forest that extends across the East, South, and Centre regions; the Guinea moist forest in the western and Adamawa regions; and the Cameroonian Highlands forests in the Northwest and Southwest regions. Those forests are crossed by several waterways, including the Sanaga River, the largest river in Cameroon (*33*–*40*; J. Thia, master’s thesis, University of Canterbury, 2014, https://www.researchgate.net/publication/272494772_The_plight_of_trees_in_disturbed_forest_conservation_of_Montane_Trees_Nigeria).

### Sample Collection

We defined a suspected case as >1 clinical signs or symptoms, including headache, asthenia, adenopathy, myalgia associated with fever, or gradually developing rashes spreading to other parts of the body, including the soles of the feet and palms of the hands. We defined a probable case as clinical manifestations without virologic confirmation but an epidemiologic link with another probable or confirmed case. A confirmed case was any case with laboratory-confirmed MPXV.

We recorded epidemiologic data, including demographic and clinical information, for all suspected cases during 1979–2022. We collected a 5-mL blood sample, vesicle swab, crust samples, or a combination of samples, from case-patients who consented to be tested. We shipped samples under a triple packaging system to the Centre Pasteur du Cameroun (CPC), which is the national reference laboratory for mpox diagnosis in Cameroon. We excluded patients from whom a sample could not be collected.

### Laboratory Confirmation of MPXV Infection

At CPC, samples were received, processed, and inactivated in the Biosafety Level 3 laboratory. We purified total DNA by using the QIAamp DNA Mini Kit (QIAGEN, https://www.qiagen.com) according to the manufacturer’s instructions. We tested purified DNA for MPXV by the generic real-time PCR Taqman assay, as previously described (*41*). For positive samples displaying a cycle threshold (Ct) value <37, we performed further genotyping by using real-time PCRs specifically targeting MPXV clade I and II (*41*).

We further amplified a subset of 8 positive samples from the 2022 outbreak that had Ct values <20 by using a PCR targeting a portion of the MPXV A-type inclusion (*ATI*) gene, according to a previously described protocol (*42*). We used a 1% green-stained agarose gel to reveal resulting amplicons, which we sent to Inqaba Biotechnical Industries (Pretoria, South Africa), a commercial service provider, for Sanger sequencing.

### Phylogenetical Analyses

We assembled newly determined sequences and corrected by using CLC Main Workbench software (QIAGEN). We aligned resulting consensus sequences by using MAFFT version 7 (https://mafft.cbrc.jp) and an extended dataset of 56 MPXV reference genomes from GenBank (Appendix Tables 1, 2). We submitted final alignments to the software-integrated Model Finder program (IQ-TREE, http://www.iqtree.org) to select the best evolutionary model based on Bayesian and Akaike information criterion. We used IQ-TREE version 1.6.12 (http://www.iqtree.org) to infer maximum-likelihood phylogenetic trees on MPXV *ATI* sequences based on the Hasegawa-Kishino-Yano plus amino acid substitution model, applying 1,000 bootstrap replicates. We submitted newly determined sequences to GenBank (accession nos. OR038717–24) (Appendix Table 2).

### Statistical Analysis and Mapping

To provide a complete picture of the epidemiology of mpox in Cameroon, we added the 4 previously documented mpox cases from Cameroon to our dataset, along with available information collected from the literature and Ministry of Health archives (*29*,*34*–*36*). We summarized sociodemographic and clinical characteristics by using frequencies for categorical variables; we used median and interquartile range (IQR) for quantitative variables. We compared PCR-confirmed cases with nonconfirmed suspected cases by using Pearson χ^2^ or Fisher exact tests for categorical variables and Wilcoxon test for quantitative variables. We used univariate logistic regression to identify factors associated with MPXV infection and estimate crude odds ratios (ORs) and 95% CIs. We were unable to infer multivariable analysis models, which failed to converge because too many data were missing (Tables 1, 2). We considered p<0.05 statistically significant and p<0.07 marginally significant. We performed all analyses in R version 4.1 (The R Foundation for Statistical Computing, https://www.r-project.org). We used Quantum GIS version 3.30.1 (QGIS, https://qgis.org) to analyze and map mpox cases by health zones and geographic data.

**Table 1:** Molecular diagnostic and epidemiologic characteristics suspected and confirmed mpox cases in a study of concurrent clade I and clade II monkeypox virus circulation, Cameroon, 1979–2022*

Epidemiologic characteristics	MPXV real-time PCR, no. (%)		Crude OR (95% CI)	p value
	Positive	Negative		
Total no. (%), n = 137	32 (23.36)	105 (76.64)		
MPXV clades				
Clade I, n = 12	12 (100.00)	NA	NA	1
Clade II, n = 18	18 (100.00)	NA	NA	
Sex				
M, n = 74	21 (28.38)	53 (71.62)	Referent	**0.142**
F, n = 62	11 (17.74)	51 (82.26)	1.84 (0.81–4.19)	
Age				
Minimum	0	0	NA	**0.075**
1st quartile	8.5	4	NA	
Median	21.5	10	NA	
Mean	21.69	15.64	1.02 (1.00–1.05)	
3rd quartile	32.25	22.5	NA	
Maximum	52	75	NA	
Age group, y				
0–10, n = 66	10 (15.15)	56 (84.85)	Referent	**0.101**
11–20, n = 24	6 (25.00)	18 (75.00)	1.87 (0.60–5.85)	
21–30, n = 18	6 (33.33)	12 (66.67)	2.8 (0.85–9.19)	
>30, n = 27	10 (37.04)	17 (62.96)	3.29 (1.17–9.24)	
Born before 1980				
Y, n = 124	27 (45.45)	97 (54.55)	Referent	**0.092**
N, n = 11	5 (21.77)	6 (78.23)	3.07 (0.86–10.88)	
Born before 2002				
Y, n = 90	16 (17.78)	74 (82.22)	Referent	**0.025**
N, n = 45	16 (35.56)	29 (64.44)	2.55 (1.13–5.77)	
Occupation				
Underage/none, n = 29	6 (20.69)	23 (79.31)	Referent	**0.067**
Pupil/student, n = 54	8 (14.81)	46 (85.19)	0.67 (0.21–2.15)	
Health worker, n = 4	2 (50.00)	2 (50.00)	3.07 (0.84–11.17)	
Farmer, n = 18	8 (44.44)	10 (55.56)	3.83 (0.44- 33.11)	
Others†, n = 14	5 (35.71)	9 (64.28)	2.13 (0.52–8.77)	
Contact with human case				
Y, n = 57	17 (29.82)	40 (70.18)	Referent	0.304
N, n = 56	10 (17.86)	46 (82.14)	0.51 (0.21–1.24)	
Unknown, n = 3	1 (33.33)	2 (66.67)	0.17 (0.10–13.86)	
Contact with animal				
Y, n = 37	12 (32.43)	25 (67.57)	Referent	0.143
N, n = 69	11 (15.94)	58 (84.06)	0.40 (0.15–1.0)	
Unknown, n = 7	2 (28.57)	5 (71.43)	0.88 (0.14–4.93)	
Contact with wild or domestic animal				
Domestic animal, n = 13	3 (23.08)	10 (76.92)	Referent	**0.06**
Wild animal, n = 13	6 (46.15)	7 (53.85)	2.86 (0.53–15.47)	
No contact, n = 69	11 (15.94)	58 (84.06)	0.63 (0.15–2.67)	
Travel history				
Y, n = 21	5 (23.81)	16 (76.19)	Referent	0.831
N, n = 96	25 (26.04)	71 (73.96)	0.89 (0.29–2.67)	
Geographic distribution				
Adamawa, n = 1	0	1 (100.00)	Referent	0.831
Centre, n = 32	11 (34.36)	21 (65.63)	0 to ∞	
East, n = 23	3 (13.04)	20 (86.96)	0 to ∞	
Far-North, n = 2	0	2 (100.00)	0 to ∞	
Littoral, n = 4	1 (25)	3 (75.00)	0 to ∞	
North, n = 1	0	1 (100.00)	0 to ∞	
North-West, n = 25	6 (24.00)	19 (76.00)	0 to ∞	
South, n = 9	1 (11.00)	8 (88.89)	0 to ∞	
South-west, n = 39	10 (25.64)	29 (74.36)	0 to ∞	
Other‡	0	1	0 to ∞	

*Bold text indicates statistical significance. Some categories might not add to 100% because of missing data. Missing data were not accounted for in the statistical analysis. MPXV, monkeypox virus; NA, not applicable; OR, odds ratio. †Others include teachers, traders, driver or motorbiker, housewife, informal, retired. ‡Equatorial Guinea.

**Table 2:** Clinical Characteristics of suspected and confirmed mpox cases in a study of concurrent clade I and clade II monkeypox virus circulation, Cameroon, 1979–2022*

Characteristics	MPXV RT-PCR, no. (%)		Crude OR (95% CI)	p value
	Positive	Negative		
Total no. (%), n = 137	32 (23.36)	105 (76.64)		
Active skin lesions				
Lesions, n = 124	29 (23.39)	95 (76.61)	Referent	0.289
No lesions, n = 10	1 (10.00)	9 (90.00)	2.75 (0.33–22.6)	
Lesion progress				
Diffuse, n = 23	5 (21.74)	18 (78.26)	Referent	0.329
Head to limbs, n = 25	4 (16.00)	21 (84.00)	0.69 (0.16–2.95)	
Limbs to head, n = 15	6 (40.00)	9 (60.00)	2.4 (0.57–10.04)	
Others, n = 24	4 (16.67)	20 (83.33)	0.72 (0.17–3.1)	
Lesions at the same stage				
Y, n = 43	13 (30.23)	30 (69.77)	Referent	0.195
N, n = 53	10 (18.87)	43 (81.13)	1.86 (0.72–4.8)	
Lesions of the same size				
Y, n = 49	13 (26.53)	36 (73.47)	Referent	0.546
N, n = 47	10 (21.28)	37 (78.72)	1.34 (0.52–3.43)	
Lesions deep				
Y, n = 42	11 (26.19)	31 (73.81)	Referent	0.767
N, n = 51	12 (23.53)	39 (76.47)	1.15 (0.45–2.97)	
Fever before rash				
Y, n = 86	22 (25.58)	64 (74.42)	Referent	**0.149**
N, n = 30	4 (13.33)	26 (86.67)	2.23 (0.7–7.12)	
Missing	6	15	NA	
Headache				
Y, n = 51	15 (29.41)	36 (70.59)	Referent	**0.1**
N, n = 61	10 (16.39)	51 (83.61)	2.13 (0.86–5.26)	
Cough				
Y, n = 38	13 (34.21)	25 (65.79)	Referent	**0.066**
N, n = 76	14 (18.42)	62 (81.58)	2.3 (0.95–5.59)	
Vomiting, nausea				
Y, n = 15	4 (26.67)	11 (73.33)	Referent	0.722
N, n = 98	22 (22.45)	76 (77.55)	1.26 (0.36–4.34)	
Chills, sweat				
Y, n = 48	18 (37.50)	30 (62.50)	Referent	**0.003**
N, n = 66	9 (13.34)	57 (86.36)	3.8 (1.52–9.48)	
Lymphadenopathy				
Y, n = 29	12 (41.38)	17 (58.62)	Referent	**0.009**
N, n =84	14 (16.37)	70 (83.33)	3.53 (1.38–9.00)	
Sore throat when swallowing				
Y, n = 28	16 (51.14)	12 (42.86)	Referent	**<0.001**
N, n = 85	10 (11.76)	75 (88.24)	10 (3.69–27.12)	
Oral ulcer				
Y, n = 18	11 (61.11)	7 (38.89)	Referent	**<0.001**
N, n = 95	15 (15.79)	80 (84.21)	8.38 (2.80–25.09)	
Itchy lesions				
Y, n = 75	19 (25.33)	56 (74.67)	Referent	0.397
N, n = 43	8 (18.60)	35 (81.40)	1.48 (0.59–3.75)	
Unknown	5	14	NA	
General fatigue				
Y, n = 62	20 (32.25)	42 (67.74)	Referent	**0.016**
N, n = 52	7 (13.46)	45 (86.54)	3.06 (1.17–7.98)	
Myalgia				
Y, n = 29	9 (31.03)	20 (68.97)	Referent	0.244
N, n = 84	17 (20.24)	67 (79.76)	1.77 (0.69–4.59)	
Unknown	6	18	NA	
Conjunctivitis				
Y, n = 14	2 (14.29)	12 (85.71)	Referent	0.385
N, n = 99	24 (24.24)	75 (75.76)	0.52 (0.11–2.49)	

*Bold text indicates statistical significance. Missing data is for patients who did not provide an answer. Unknown is for persons who replied that they did not know. Some categories might not add to 100% because of missing data. Missing data were not accounted for in the statistical analysis. MPXV, monkeypox virus; NA, not applicable; OR, odds ratio.

### Ethics

Sample collection and laboratory analyses were conducted within the framework of the Cameroon national surveillance program. Under that program, we obtained written or oral informed consent from all persons with suspected mpox after we provided detailed information and explanations of the sampling purpose. We obtained informed consent from parents or recognized guardians for persons <15 years of age.

## Results

Within the mpox surveillance system in Cameroon, during 1979­–2022, we identified 137 suspected mpox cases, including 74 (54.41%) among male and 62 (45.59%) among female persons; 1 case had missing data for sex (Table 1). The median age of case-patients was 11 years (range 2 weeks–75 years; IQR 4–27 years); nearly half (48.18%) were <10 years of age (Table 1).

### Molecular Diagnostic Results

**Figure 1:**
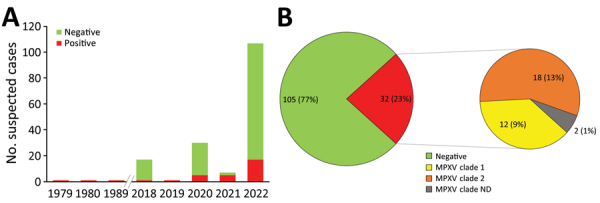
Mpox cases in a study of concurrent clade I and clade II MPXV circulation, Cameroon, 1979–2022. A) Epidemiologic curve of 137 suspected mpox cases. A 30-year gap occurred between the first 3 reported mpox cases and the consecutive cases since 2018, demonstrating increased surveillance in the country. The peak in 2022 corresponds to the worldwide alert raised on mpox, which led to enhanced mpox surveillance in Cameroon. B) Mpox genotyping results showing both clade I and clade II MPXV were identified. MPXV, monkeypox virus; ND, not determined.

Mpox virus generic PCR showed 32 (23.36%) laboratory-confirmed mpox cases of 137 patients tested during 1979­–2022 in Cameroon (Table 1; Figure 1, panel A; Appendix Table 3). Before 2018, only 3 sporadic cases were confirmed as human MPXV infection. After a 30-year gap without reported mpox cases, the surveillance system continuously identified new mpox cases during 2018–2022. Among suspected cases, only 1 was found in 2018 and 1 in 2019. In 2020 and 2021, 5 laboratory-confirmed cases were recorded each year. During 2022, mpox cases dramatically increased to 17 confirmed cases among 84 suspected cases (Figure 1, panel A; Appendix Table 3).

Genotyping of real-time PCR results identified 12 (9%) patients infected with MPXV clade I and 18 (13%) infected with MPXV clade II among 137 suspected cases; 2 (1%) historic confirmed cases lacked clade determination results (Table 1; Figure 1, panel B; Appendix Tables 2, 3). Among all laboratory-confirmed cases, only 1 death was recorded, in a patient infected with MPXV clade II. Ministry of Health investigation records indicated 2 additional patient deaths among persons with typical mpox clinical manifestations who were epidemiologically linked to 2 confirmed case-patients infected with a clade II MPXV strain. However, no specimens were collected before death; thus, we considered those probable cases. Including the probable cases, the overall case-fatality ratio (CFR) in Cameroon was 2.2% (3/139) among confirmed and suspected cases, and all deaths were associated with viral clade II.

### Epidemiology and Clinical Characteristics of Confirmed Mpox Cases

Univariable analysis revealed no statistically significant difference in increased likelihood of infection by sex: 21/74 (28.38%) male and 11/62 (17.74%) female persons had confirmed MPXV infection (Table 1). MPXV-confirmed case-patients had a median age of 21.5 years (range 2 weeks–52 years; IQR 8.5–32.25 years). MPXV infection was more prevalent among adults >20 years of age; in all, 35.56% had confirmed MPXV infection, compared with 17.78% among younger MPXV-confirmed case-patients (p = 0.025). However, we saw no statistically significant difference for adults born before 1980 than for the rest of the population (p = 0.092). Larger datasets would be needed to confirm the observed trend.

MPXV infection was mostly associated with occupational activities involved in farming (OR 3.83, 95% CI 0.44–33.11) (Table 1). Similarly, potential nosocomial transmission was identified in health workers (OR 3.07, 95% CI 0.84–11.17). Other activities, including teaching, trading, or driving, when considered together, also appeared to be potential risk activities for secondary MPXV transmission (OR 2.13, 95% CI 0.52–8.77). However, we found no association for secondary transmission in the 29.82% of MPXV-confirmed cases reporting past contact with persons who had mpox-like clinical signs (Table 1). Because mpox is typically zoonotic, we also assessed antecedent of animal exposures. We observed no association with unspecified animal contacts but observed a higher risk among confirmed cases (6/13 [46.15%]) who reported contact with wild animals (OR 2.86, 95% CI 0.53–15.47) compared with persons reporting contact with domestic animals or having no contact with animals (Table 1). Among wild animal contact, study participants frequently mentioned squirrels, bats, caterpillars, pangolins, rats, porcupines, and monkeys.

**Figure 2:**
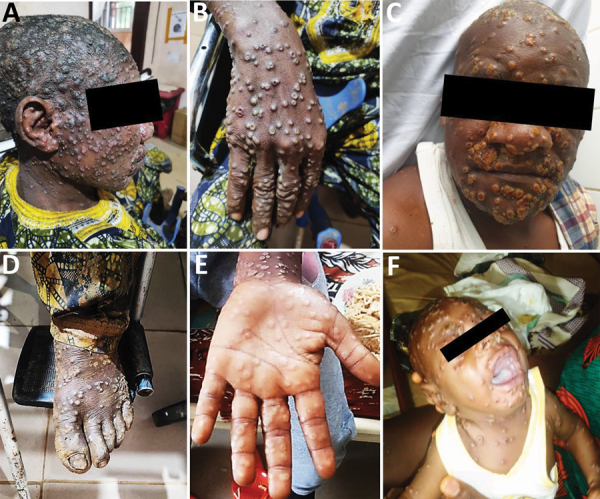
Maculopapular lesions in mpox patients from a study of concurrent clade I and clade II monkeypox virus circulation, Cameroon, 1979–2022. A–E) Deep maculopapular lesions of different sizes spread from the head (A, C) to hands (B) and diffuse to the soles of the feet (D) the palm of the hand (E). F) Lesions, including oral lesions and mouth ulcers, in a 3-month-old male baby.

As expected from the case definition criterium requiring skin rashes, almost all (124/137 [90.5%]) MPXV-suspected cases had active skin lesions (Table 2; Figure 2; Appendix Table 3). However, we observed no specific difference for lesion progress, deepness, size, or stage among MPXV-confirmed cases compared with MPXV-negative persons (Table 2). Maculopapular lesions were more prevalent in confirmed cases who had lesions on their palms and soles (Figure 2). Clinical data identified cough (OR 2.3, 95% CI 0.95–5.59), chills or sweat (OR 3.8, 95% CI 1.52–9.48), lymphadenopathy (OR 3.53, 95% CI 1.38–9.00), sore throat when swallowing (OR 10, 95% CI 3.69–27.12), mouth ulcers (OR 8.38, 95% CI 2.8–25.09), and general fatigue (OR 3.06, 95% CI 1.17–7.98) as potential symptoms associated with MPXV infection in Cameroon (Table 2; Figure 2). Among all suspected case-patients, ≈26% who reported experiencing fever before skin rashes developed were confirmed for MPXV infection, but we saw no difference between confirmed cases with or without fever. In addition, MPXV-confirmed or -negative cases did not experience differences in headache (Table 2). We noted little difference in clinical severity in cases infected with clade I compared with those infected with clade II (Appendix Table 4). The same was true for the exposure route; we found no association between zoonotic or human-to-human transmission and a specific infecting viral clade (Appendix Table 4). However, because considerable data were missing (Tables 1, 2) we were unable to perform a multivariable analysis. Therefore, concluding interpretations of the epidemiologic and clinical features of mpox infection in Cameroon are difficult to draw.

### Geographic and Phylogenetic Analysis

**Figure 3:**
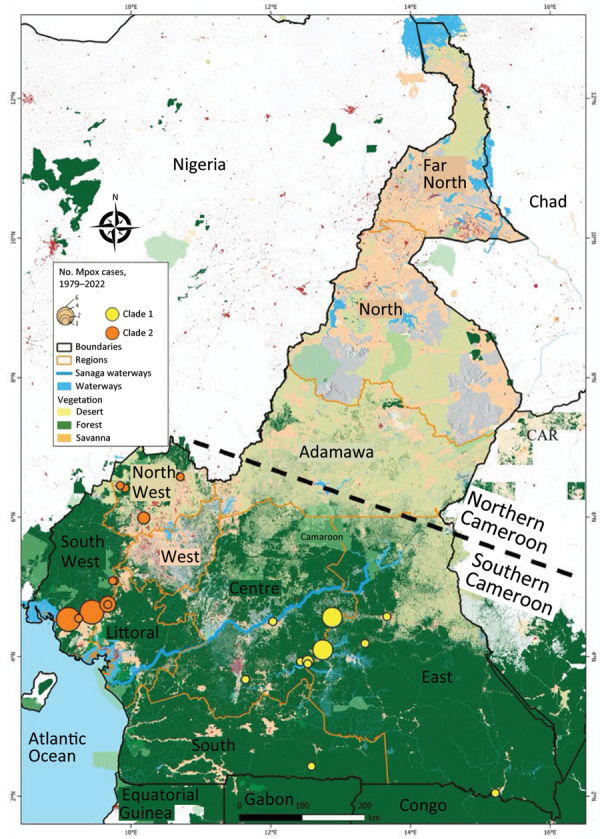
Geographic distribution of confirmed mpox cases and clades in a study of concurrent clade I and clade II monkeypox virus circulation, Cameroon, 1979–2022. A total of 137 suspected mpox cases were reported in the framework of the mpox surveillance system, among which 37 were PCR-confirmed for monkeypox virus infection. Clade I (12 cases) and clade II (18 cases) viral strains were identified circulating in the country. We noted a clear geographic segregation between the Centre, South, and East regions where only clade I (yellow dots) was reported, and the Northwest, and Southwest regions where only clade II (orange dots) was found. The size of each dot is proportional to the number of confirmed cases on the map. The map was designed by using Quantum GIS version 3.30.1 (QGIS, https://qgis.org). CAR, Central African Republic.

Reported suspected mpox cases originated from 8 administrative regions of Cameroon (Table 1; Figure 3). Most (97.08%) suspected cases were reported from the southern part of the country where all confirmed cases also originated. In particular, 1 (3.13%) case was confirmed in Littoral, 1 (3.13%) in the South, 3 (9.38%) in the East, 6 (18.75%) in the Northwest, 10 (31.25%) in the Southwest, and 11 (34.88%) in the Centre regions (Table 1; Figure 3; Appendix Table 3). Of note, a unique case confirmed in the Littoral region was originally from the Southwest and sought healthcare in Littoral. Genotyping of real-time PCR revealed that all clade I MPXV infections were confirmed in patients from the Centre, South, and East regions; all but 1 of clade II MPXV samples were recovered from patients from the Littoral, Northwest, and Southwest regions. Indeed, a clade II MPXV detected in the Centre region was an internally displaced person (IDP) originally from the Northwest region (Table 1; Appendix Table 3). The distribution of mpox cases points toward geographic segregation of the 2 viral clades in Cameroon. Those findings indicate a strong geographic association of MPXV genotypes in southern Cameroon, and that MPXV clade II is associated with the western part and the clade I with the eastern part of the country.

**Figure 4:**
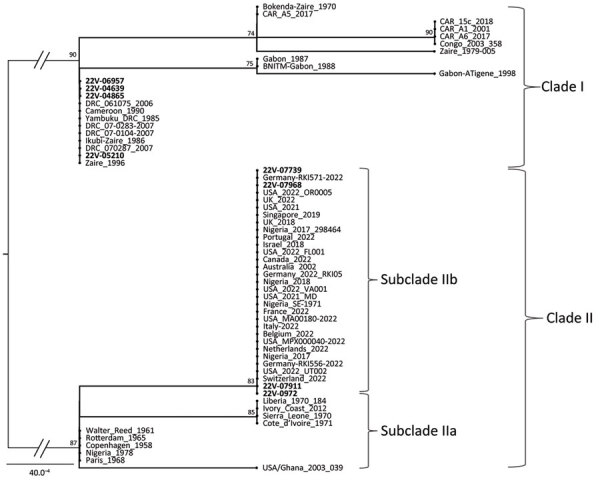
Maximum-likelihood phylogenetic tree of sequences in a study of concurrent clade I and clade II monkeypox virus circulation, Cameroon, 1979–2022. The tree is based on the Hasegawa-Kishino-Yano model inferred from a 942-bp fragment of the *ATI* gene, including 8 virus sequences from Cameroon generated in this work (bold text) and 55 reference sequences from GenBank. The tree with the highest log likelihood (−1,340.35) is shown. To test the robustness of the tree topology, 1,000 bootstrap replicates were performed. For a better display of the tree, the size of the 2 main midpoint rooted branches (represented in gray) that support the differentiation of the 2 monkeypox virus clades have been divided by half. Mpox strains from Cameroon are closely related to clades I and II, especially clade IIb for which a highlighted link to the ongoing global mpox epidemic is noted. Scale bar indicates number of substitutions per site. CAR, Central African Republic; DRC, Democratic Republic of the Congo; USA, United States.

We obtained partial MPXV *ATI* gene sequences from 8 mpox-confirmed cases from 4 regions of Cameroon. We derived the newly determined sequences from samples collected in the Northwest (CPC code 22V-0972), Southwest (CPC codes 22V-07739, 22V-07911, 22V-07968), Centre (CPC codes 22V-05210, 22V-04865, 22V-4639), and South (CPC code 22V-6957) regions. Maximum-likelihood phylogenetic analysis of the 942 nt consensus sequences, including reference sequences (Appendix Tables 1–3), revealed that the 8 MPXV genomes from Cameroon segregated into clade I and clade II. As expected from the geographic association of MPXV isolates we report, MPXV clade I from the Centre and South regions grouped reliably with reference counterparts previously reported from countries in Central Africa, and clade II sequences from the Northwest and Southwest regions grouped consistently with strains from West Africa (Figure 4). Clade II strains from Cameroon clustered reliably within subclade IIb with 83% bootstrap support (Figure 4). Altogether, genotypic and phylogenetic analysis confirmed the concurrent circulation of both MPXV clades I and II in Cameroon with a striking geographic segregation.

## Discussion

We examined the clinical, epidemiologic, and molecular patterns of MPXV infection in Cameroon over a 44-year period (1979–2022) as part of mpox surveillance in the country. During 1979–2022, a total of 137 persons were suspected of having mpox, and 32 were confirmed to be MPXV infected. Three persons died (CFR 2.2%) and death was associated with MPXV clade II. That CRF is much lower than those reported in previous studies of MPXV clade I that showed CFRs of 7%–10% (*13*,*43*). Overall, CFRs are lower among patients infected with clade II, including in the 2022 global outbreak settings (*4*,*25*). We were not able to collect information on potential underlying conditions of case-patients to determine whether immunocompromising conditions contributed to death, which would have worsened the clinical disease manifestations, as highlighted by others (*44*). In addition, fatal cases associated with clade I potentially escaped the national surveillance system in Cameroon, which is new and still being improved.

We found that both primary zoonotic and secondary human-to-human MPXV transmission occurs in Cameroon, including nosocomial transmission affecting health workers. Our results are consistent with reports describing secondary transmission chains, including intrafamilial transmission and occupational transmission through trade, transportation, hunting, and healthcare in endemic countries (*24*,*43*,*45*,*46*). This study highlights a common MPXV acquisition pathway in endemic countries, interspecies transmission, and wild animals are presumed reservoirs of the virus (*31*,*32*,*47*). Distinguishing between primary and secondary transmission is difficult because both could occur. Additional data and further investigations are required to clearly understand the underlying drivers of MPXV transmission in Cameroon. 

A limitation of this study is our inability to perform more precise analyses to determine the characteristics independently describing the mpox epidemiology in Cameroon. Because the current surveillance system is still handwritten and forms are often incompletely filled, data are missing, as is common in paper-based data collection systems (*48*).

Since 1979, MPXV infections in Cameroon have occurred in 6 of the 10 administrative divisions of the country: Centre, South, East, Littoral, Northwest, and Southwest. All those administrative divisions are in the southern part of the country, which is a forested area encompassed by the lower montane forest of Guinea and the tropical rainforest of the Congo Basin, a favorable ecosystem for potential wildlife hosts. In contrast, northern Cameroon, a dry Sahelian and savannah zone, seems unlikely to be conducive to MPXV transmission because no cases have been confirmed in this region. That ecosystem is probably not suitable for MPXV reservoirs due to the dry environment. In most endemic countries, including Sierra Leone, Nigeria, Liberia, Central African Republic, and the Democratic Republic of the Congo, mpox cases mainly have been reported from forested areas (*24*,*25*,*46*). Most MPXV-confirmed cases in our study originated from the Centre (34 [38%]) and Southwest (31 [25%]) regions, which are the 2 most affected areas in the country. The Northwest region was the third (18 [75%] cases) most affected region. The Northwest and Southwest regions have been most seriously affected by civil unrest since 2017. That civil unrest has increased the number of IDPs in the country, and IDPs often move to different regions and neighboring countries. Furthermore, that situation has greatly increased human contact with wildlife as IDPs seek refuge in makeshift camps in the forest. By living in overlapping natural habitats of wild animals and potential MPXV reservoirs, populations of the Southwest and Northwest regions are under increased threat of zoonotic MPXV acquisition. Indeed, in Africa, civil unrest often leads to increases in mpox cases, and risk for any zoonotic disease is common (*4*,*49*). In several endemic countries, mpox outbreaks in the context of armed conflicts or massive population movements are a typical epidemiologic feature, and those conditions are usually associated with inefficient disease surveillance and control (*4*,*49*).

Genotypic and phylogenetic analyses revealed that both clade I and clade II are concurrently circulating in Cameroon and that a clear geographic segregation appears between the 2 clades. Circulation of both MPXV clades in Cameroon was previously reported in 2 published MPXV sequences from Cameroon (*6*,*29*). However, this study builds on those findings and provides more samples to further confirm that clades I and II concurrently circulate in a single country, a unique feature in MPXV epidemiology. 

The geographic segregation of the clades is more perceptible in clade II case 21V-04877 in the Centre region. An epidemiologic investigation revealed that the case-patient was an IDP originating from the Northwest region, where MPXV clade II is endemic. The geographic segregation observed between MPXV strains circulating in Cameroon can be attributed to the natural barriers that potential animal reservoirs might not be able to cross between the Centre, East, and South regions, covered by the Congo Basin tropical forest, and the Northwest and Southwest regions, covered by lower montane moist forest of Guinea (*38*,*40*). Indeed, the Sanaga River, which is the largest river in the country, and the Cameroon highlands region sharply separate the 2 geographic areas into tropical moist forest ecoregions. The Cross-Sanaga-Bioko coastal forests lie to the north between the Sanaga River and the Cross River of Nigeria, and the Atlantic Equatorial coastal forests extends south of the river through southwestern Cameroon and other neighboring countries of central Africa (*38*,*39*). Alternatively, the 2 ecologic environments potentially host different reservoirs. Several studies aimed to identify presumed MPXV reservoirs (*31*,*33*,*47*), but none have emphasized the potential of 2 distinct reservoirs that could be specific to a given ecosystem. Furthermore, MPXV circulation in humans in Cameroon after decades of absence might have resulted from movements of human populations, reservoir hosts, or both from endemic reservoirs in neighboring countries as armed conflicts intensified cross-border movements since 2017. That hypothesis is supported by the clustering of newly sequenced MPXV strains with counterparts originating from neighboring countries that have no physical barrier with the eastern and western parts of Cameroon but have long terrestrial borders.

In summary, this study provides detailed insight into the mpox epidemic in Cameroon during a 44-year period. The epidemiology of mpox in Cameroon involves both primary and secondary transmission. Segregated clade I and II virus strains concurrently circulate, suggesting potential existence of distinct viral reservoirs and cross-border circulation of MPXV. This study can inform the design, optimization, and evaluation of public health interventions for monitoring and controlling mpox in Cameroon and other countries in Africa with similar epidemiologic settings.

Appendix Additional information on concurrent clade I and clade II monkeypox virus circulation, Cameroon, 1979–2022.
