# Supplementary material for: Concurrent Clade I and Clade II Monkeypox Virus Circulation, Cameroon, 1979–2022
Source: Emerg Infect Dis. 2024 Mar;30(3):432–43. doi: 10.3201/eid3003.230861 (PMC10902553; doi:10.3201/eid3003.230861)
Supplement: Appendix — Additional information on concurrent clade I and clade II monkeypox virus circulation, Cameroon, 1979–2022. [file 23-0861-Techapp-s1.pdf]

Article DOI: https://doi.org/10.3201/eid3003.230861

EID cannot ensure accessibility for supplementary materials supplied by authors. Readers who have difficulty accessing supplementary content should contact the authors for assistance.

Concurrent Clade I and Clade II Monkeypox Virus Circulation, Cameroon, 1979–2022

Appendix

Appendix Table 1. Public monkeypox virus genomes used for phylogenetic analysis*

No.	Country	Year	Original name	Name on tree	Host	Accession no.	Clade	Sub-clade	Lineage
1	Cameroon	1989	Cameroon-1990	Cameroon_1990	Human	KJ642618	I	I	1
2	Gabon	1987	Gabon 1988	Gabon_1988	Human	KJ642619	I	I	1
3	Gabon	1988	BNITM-Gabon1988	BNITM-Gabon_1988	Human	OP498046	I	I	1
4	DRC	1970	Congo_8	Bokenda-Zaire_1970	Human	KJ642613	I	I	2
5	DRC	2003	Congo_2003_358	Congo_2003_358	Human	DQ011154	I	I	2
6	CAR	2018	015c_contig_SPADES	CAR_15c_2018	Human	MN702448	I	I	2
7	CAR	2001	A1_contig_SPADES	CAR_A1_2001	Human	MN702453	I	I	2
8	CAR	2017	A6_contig_SPADES	CAR_A6_2017	Human	MN702451	I	I	2
9	CAR	2018	A5_contig_SPADES	CAR_A5_2017	Human	MN702444	I	I	2
10	DRC	1985	Yambuku_DRC_1985	Yambuku_DRC_1985	Squirrel	KP849471	I	I	3
11	DRC	2006	DRC_06–1074	DRC_06–1075–2006	Human	JX878411	I	I	3
12	DRC	2007	DRC_07–0286	DRC_07–0287–2007	Human	JX878422	I	I	3
13	DRC	2007	DRC 07–0283	DRC_07–0283–2007	Human	JX878420	I	I	3
14	DRC	1986	Ikubi	Ikubi-Zaire_1986	Human	KJ642612	I	I	4
15	DRC	1996	Monkeypox virus	Zaire_1996	Human	NC_003310	I	I	4
16	DRC	1979	Zaire 1979–005	Zaire_1979–005	Human	HM172544	I	I	
17	Gabon	1998	A-type inclusion body protein gene	Gabon-ATIgene_1998	Human	U84504	I	I	
18	DRC	2007	DRC 07–0104	DRC_07–0104–2007	Human	JX878417	I	I	5
19	USA	2022	hMPX/USA/CA-LACPHL-MA00154/2022	USA_2022_MA00180	Human	OP580587	II	IIa	
20	USA	1971	Nigeria-SE-1970	Nigeria_SE_1971	Human	KJ642617	II	IIa	
21	Nigeria	2017	MPXV_Nig_2017_298464	Nigeria_2017_298464	Human	MG693724	II	IIa	
22	USA	2022	MPXV/Human/USA/CA-CDPH-MPX000040/2022	USA_2022_MPX000040	Human	OP587264	II	IIa	
23	Liberia	1970	Liberia_1970_184	Liberia_1970_185	Human	DQ011156	II	IIa	
24	Cote d’Ivoire	1970	Cote d'Ivoire_1971	Ivory_Coast_1971	Human	KP849470	II	IIa	
25	Sierra Leone	1970	Sierra Leone	Sierra_Leone_1970	Human	AY741551	II	IIa	
26	USA	1961	MPXV-WRAIR7–61	Walter_Reed_1961	Human	AY603973	II	IIa	
27	USA	2003	USA_2003_039	USA/Ghana_2003	Human	DQ011157	II	IIa	
28	France	1968	PCH	Paris_1968	Human	KJ642616	II	IIa	
29	Netherlands	1965	UTC	Rotterdam_1965	Human	KJ642614	II	IIa	
30	Denmark	1958	COP-58	Copenhagen_1958	Human	AY753185	II	IIa	
31	Nigeria	1978	W-Nigeria	Nigeria_1978	Human	KJ642615	II	IIa	
32	Cote d’Ivoire	2012	Ivory Coast 2012	Ivory_Coast_2012	Human	KJ136820	II	IIa	
33	Germany	2022	MPXV/Germany/2022/RKI571	Germany-RKI571_2022	Human	OP764629	II	IIa	
34	Germany	2022	MPXV/Germany/2022/RKI556	Germany-RKI556_2022	Human	OP764614	II	IIa	
35	USA	2022	MPXV_USA_2022_OR0005	USA_2022_OR0005	Human	OP752116	II	IIa	
36	Nigeria	2017	MPXV-M2957_Lagos	Nigeria_2017	Human	MT903338.1	II	IIb	A
37	Nigeria	2018	MPXV-M3021_Delta	Nigeria_2018	Human	MT903339.1	II	IIb	A
38	Singapore	2019	MPXV-Singapore	Singapore_2019	Human	MT903342.1	II	IIb	A.1
39	UK	2018	MPXV-UK_P2	UK_2018	Human	MT903344.1	II	IIb	A.1

No.	Country	Year	Original name	Name on tree	Host	Accession no.	Clade	Sub-clade	Lineage
40	Switzerland	2022	MPXV-CH-38134631/2022	Switzerland_2022	Human	ON595760.2	II	IIb	B.1
41	Italy	2022	INMI-Pt1	Italy-2022	Human	ON614676.1	II	IIb	B.1
42	Netherlands	2022	MPXV_2022_NL001	Netherlands_2022	Human	ON615424.1	II	IIb	B.1
43	UK	2022	MPXV_UK_2022_3	UK_2022	Human	ON619837.2	II	IIb	B.1
44	Belgium	2022	MPX/UZ_REGA_1/Belgium/2022	Belgium_2022	Human	ON622712.1	II	IIb	B.1
45	France	2022	MPXV_FR_HCL0001_2022	France_2022	Human	ON622722.2	II	IIb	B.1
46	Australia	2022	MPxV/VIDRL01/2022	Australia_2022	Human	ON631963.1	II	IIb	B.1
47	Portugal	2022	Monkeypox/PT0027/2022	Portugal_2022	Human	ON649710.1	II	IIb	B.1
48	USA	2022	MPXV_USA_2022_FL001	USA_2022_FL001	Human	ON674051.1	II	IIb	A.2
49	USA	2022	MPXV_USA_2022_VA001	USA_2022_VA001	Human	ON675438.1	II	IIb	A.2
50	USA	2022	MPXV_USA_2022_UT002	USA_2022_UT002	Human	ON676706.1	II	IIb	B.1
51	USA	2021	MPXV_USA_2021_TX	USA_2021_TX	Human	ON676707.1	II	IIb	A.2
52	USA	2021	MPXV_USA_2021_MD	USA_2021_MD	Human	ON676708.1	II	IIb	A.1.1
53	Germany	2022	MPXV/Germany/2022/RKI05	Germany_2022_RKI05	Human	ON682264.4	II	IIb	B.1
54	Canada	2022	MPX/2022/Canada/AB1	Canada_2022	Human	ON736420.1	II	IIb	B.1
55	Israel	2018	Israel_2018	Israel_2018	Human	MN648051.1	II	IIb	A.1

*Available in GenBank (https://www.ncbi.nlm.nih.gov/genbank). CAR, Central African Republic; DRC, Democratic Republic of the Congo; UK, United Kingdom; USA, United States of America.

Appendix Table 2. Epidemiologic and molecular features of monkeypox virus sequences obtained from Cameroon

No.	Country	Age, y/sex	Collection year	Original SEQ_ID	SEQ_ID on tree	Host	Isolation source region/health district	MPXV clade	Accession no.
1	Cameroon	36/F	2022	22V-0972	22V-0972	Human	South-West/Benakuma	II	OR038717
2	Cameroon	10/M	2022	22V-06957	22V-06957	Human	South/Djoum	I	OR038718
3	Cameroon	6/F	2022	22V-04639	22V-04639	Human	Centre/Ayos	I	OR038719
4	Cameroon	20/M	2022	22V-04865	22V-04865	Human	Centre/Ayos	I	OR038720
5	Cameroon	11/F	2022	22V-07739	22V-07739	Human	South-West/Kumba South	II	OR038721
6	Cameroon	52/M	2022	22V-07911	22V-07911	Human	South-West/Deido-Tombel	II	OR038722
7	Cameroon	40/M	2022	22V-05210	22V-05210	Human	Centre/Ayos	I	OR038723
8	Cameroon	43/M	2022	22V-07968	22V-07968	Human	South-West/Tombel	II	OR038724

Appendix Table 3. Epidemiologic information of mpox confirmed cases in Cameroon, 1979–2022

									Date	
No.	CPC code	Country	Region	Age, y/sex	Patient progress	MPXV PCR	MPXV clade	VZV PCR	Disease onset	Sampling
1	UNK	Cameroon	Centre	4/F	Unk	+	UNK	UNK	UNK	1979 Jan 1
2	UNK	Cameroon	East	3/M	Unk	+	UNK	UNK	UNK	1980 Jan 1
3	UNK	Cameroon	Centre	7/M	Alive	+	I	UNK	1989 Dec 4	1989 Dec 11
4	18V-04552	Cameroon	Northwest	20/M	Alive	+	II	UNK	UNK	2018 May 4
5	19V-06906	Cameroon	Southwest	14/M	Alive	+	II	UNK	2019 Sep 7	2019 Sep 19
6	20V-00071	Cameroon	Centre	27/F	Alive	+	I	–	2020 Jan 3	2020 Jan 4
7	20V-02998	Cameroon	East	36/M	Alive	+	I	–	2020 Mar 14	2020 Mar 24
8	20V-14349	Cameroon	East	31/F	Alive	+	I	–	2020 Aug 26	2020 Sep 6
9	20V-14644	Cameroon	Centre	46/M	Alive	+	I	–	2020 Sep 16	2020 Sep 23
10	20V-14645	Cameroon	Centre	43/F	Alive	+	I	–	2020 Sep 18	2020 Sep 23
11	21V-04877	Cameroon	Centre	30/F	Alive	+	II	UNK	UNK	2021 Jul 22
12	21V-09233	Cameroon	Centre	11/M	Alive	+	I	–	2021 Dec 3	2021 Dec 13
13	21V-09234	Cameroon	Centre	8/M	Alive	+	I	–	2021 Nov 27	2021 Dec 15

No.	CPC code	Country	Region	Age, y/sex	Patient progress	MPXV PCR	MPXV clade	VZV PCR	Date	
									Disease onset	Sampling
14	21V-09565	Cameroon	Southwest	0/M	Alive	+	II	–	2021 Dec 22	2021 Dec 29
15	21V-09567	Cameroon	Southwest	31/M	Dead	+	II	–	2021 Dec 23	2021 Dec 29
16	22V-00302	Cameroon	Southwest	27/F	Alive	+	II	–	2021 Dec 27	2022 Jan 27
17	22V-00972	Cameroon	Northwest	36/F	Alive	+	II	–	UNK	2022 Feb 16
18	22V-00973	Cameroon	Northwest	1/F	Alive	+	II	–	UNK	2022 Feb 16
19	22V-04639	Cameroon	Centre	10/M	Alive	+	I	–	2022 Jun 9	2022 Jun 13
20	22V-04865	Cameroon	Centre	6/F	Alive	+	I	–	2022 Jun 20	2022 Jun 21
21	22V-05210	Cameroon	Centre	20/M	Alive	+	I	–	2022 Jun 28	2022 Jul 4
22	22V-06957	Cameroon	South	11/F	Alive	+	I	–	2022 Sep 5	2022 Sep 7
23	22V-07739	Cameroon	Southwest	52/M	Alive	+	II	–	2022 Sep 19	2022 Oct 6
24	22V-07911	Cameroon	Littoral	40/M	Alive	+	II	–	2022 Sep 25	2022 Oct 11
25	22V-07968	Cameroon	Southwest	43/M	Alive	+	II	–	UNK	2022 Oct 13
26	22V-08077	Cameroon	Southwest	23/F	Alive	+	II	–	2022 Oct 12	2022 Oct 14
27	22V-08300	Cameroon	Northwest	28/M	Alive	+	II	–	2022 Oct 14	2022 Oct 22
28	22V-08501	Cameroon	Southwest	23/M	Alive	+	II	–	UNK	2022 Oct 27
29	22V-08502	Cameroon	Southwest	18/M	Alive	+	II	–	UNK	2022 Oct 27
30	22V-08503	Cameroon	Southwest	0/M	Alive	+	II	–	UNK	2022 Oct 26
31	22V-09327	Cameroon	Northwest	1/M	Alive	+	II	–	2022 Nov 3	2022 Nov 8
32	22V-09403	Cameroon	Northwest	43/M	Alive	+	II	–	2022 Nov 2	2022 Nov 11

*CPC, Centre Pasteur du Cameroun; MPXV, monkeypox virus; UNK, unknown; ZVZ, +, positive; –, negative

Appendix Table 4. Severity of monkeypox virus clades in 30 confirmed cases in Cameroon from 1979 to 2022

Clinical Characteristics	MPXV RT-PCR, no. (%)		Crude OR (95% CI)	p value
	Clade I, n = 12 (40.00)	Clade II, n = 18 (60.00)		
Active Skin Lesions				
Y	12 (100.00)	17 (94.44)	1 (Referent)	0.995
N	0 (0.00)	1 (5.56)	0 (0.00–58.5)	
Missing	0	0	NA	
Lesion progress				
Diffuse	1 (14.29)	4 (33.33)	1 (Referent)	0.066
Head to limbs	0 (00.00)	4 (33.33)	0.00 (0 to ∞)	
Limbs to head	3 (42.86)	3 (25.00)	4.00 (0.27–60.32)	
Others	3 (42.86)	1 (8.33)	12.00 (0.51–280.09)	
Missing	5	6	NA	
Lesions at same stage				
Y	7 (70.00)	6 (46.15)	1 (Referent)	0.249
N	3 (30.00)	7 (53.85)	2.72 (0.48–15.47)	
Missing	2	5	NA	
Lesions of same size				
Y	8 (80.00)	5 (38.46)	1 (Referent)	0.057
N	2 (20.00)	8 (61.54)	6.4 (0.95–43.23)	
Missing	2	5	NA	
Lesions deep				
Y	4 (40.00)	7 (53.85)	1 (Referent)	0.511
N	6 (60.00)	6 (46.15)	0.57 (0.11–3.04)	
Missing	2	5	NA	
Fever before rash				
Y	12 (100.00)	10 (71.43)	1 (Referent)	0.995
N	0 (0.00)	4 (28.57)	0.00 (0.00–1.61)	
Missing	0	4	NA	
Headache				
Y	9 (75.00)	6 (53.85)	1 (Referent)	0.149
N	3 (25.00)	7 (46.15)	3.50 (0.64–19.19)	
Missing	0	5	NA	
Cough				
Y	7 (58.33)	6 (40.00)	1 (Referent)	0.346
N	5 (41.67)	9 (60.00)	2.1 (0.45–9.84)	

Clinical Characteristics	MPXV RT-PCR, no. (%) Clade I, n = 12 (40.00)	Clade II, n = 18 (60.00)	Crude OR (95% CI)	p value
Missing	0	3	NA	
Vomiting, nausea				
Y	2 (16.67)	2 (14.29)	1 (Referent)	0.867
N	10 (83.33)	12 (85.71)	1.20 (0.14–10.12)	
Missing	0	4	NA	
Chills, sweat				
Y	8 (66.67)	10 (66.67)	1 (Referent)	1.000
N	4 (33.33)	5 (33.33)	1.00 (0.20–5.00)	
Missing	0	3	NA	
Lymphadenopathy				
Y	8 (66.67)	10 (71.43)	1 (Referent)	0.059
N	4 (33.33)	4 (28.57)	5.00 (0.94–26.53)	
Missing	0	4	NA	
Sore throat when swallowing				
Y	9 (75.00)	7 (50.00)	1 (Referent)	0.199
N	3 (25.00)	7 (50.00)	3.00 (0.56–16.01)	
Missing	0	4	NA	
Oral ulcer				
Y	8 (66.67)	3 (21.43)	1 (Referent)	**0.026**
N	4 (33.33)	11 (78.57)	7.33 (1.27–42.29)	
Missing	0	4	NA	
Itchy lesions				
Y	11 (91.67)	8 (53.33)	1 (Referent)	**0.052**
N	1 (8.33)	7 (46.67)	9.62 (0.98–94.54)	
Missing	0	3	NA	
General fatigue				
Y	10 (83.33)	10 (66.67)	1 (Referent)	0.334
N	2 (16.67)	5 (33.33)	2.5 (0.39–16.05)	
Missing	0	3	NA	
Myalgia				
Y	5 (41.67)	4 (28.57)	1 (Referent)	0.486
N	7 (58.33)	10 (71.43)	1.79 (0.35–9.13)	
Missing	0	4	NA	
Conjunctivitis				
Y	1 (8.33)	1 (7.14)	1 (Referent)	0.910
N	11 (91.67)	13 (92.86)	1.18 (0.07–21.17)	
Missing	0	4	NA	
Contact with human case				
Y	7 (58.33)	10 (62.50)	1 (Referent)	0.510
N	5 (41.67)	5 (31.25)	1.42 (0.30–6.88)	
Unknown	0 (0.00)	1 (6.25)	0.00 (0 to ∞)	
Missing	0	2	NA	
Contact with animal				
Y	7 (58.33)	5 (38.46)	1 (Referent)	0.570
N	4 (33.33)	7 (53.85)	0.40 (0.08–2.19)	
Unknown	1 (8.33)	1 (7.69)	0.71 (0.04–14.35)	
Missing	0	5	NA	
Contact with wild or domestic animal				
Domestic animal	2 (20.00)	1 (10.00)	1 (Referent)	0.397
Wild animal	4 (40.00)	2 (20.00)	1.00 (0.05–18.91)	
No contact	4 (40.00)	7 (70.00)	0.29 (0.02–4.24)	
Missing	2	8	NA	

*Bold text indicates statistical significance. MPXV, monkeypox virus; NA, not applicable; OR, odds ratio; RT-PCR, reverse transcription PCR.
